# Dual time point imaging of FDG PET/CT in a tuberculous spondylodiscitis

**DOI:** 10.2349/biij.6.2.e18

**Published:** 2010-04-01

**Authors:** HR Abdul, N Abdul, AJ Nordin

**Affiliations:** 1 Discipline of Medical Radiations, School of Medical Sciences, RMIT University, Melbourne, Victoria, Australia.; 2 Medical Imaging Department, Faculty of Health Sciences, University Technology MARA, Selangor, Malaysia.; 3 Radiology Department, Serdang Hospital, Selangor, Malaysia.; 4 Nuclear Imaging Unit, Faculty of Medicine and Health Sciences, University Putra Malaysia, Selangor, Malaysia.

**Keywords:** Tuberculosis, Positron Emission Tomography/Computed Tomography, FDG, Dual Time Point Imaging

## Abstract

Dual Time Point Imaging (DTPI) technique is a specialised protocol adopted in ^18^F-Fluorodeoxyglucose (FDG) Positron Emission Tomography (PET) imaging. This technique is claimed to be useful in differentiating malignant and infective lesions. The authors adopted this technique in a patient diagnosed with tuberculous spondylodiscitis and psoas abscess which demonstrated higher Maximum Standardized Uptake Value (SUVmax) during initial scans as compared with those obtained on delayed scans. The SUVmax changes between the two time points are believed to be a valuable finding for chronic granulomatous infective lesions such as tuberculosis.

## INTRODUCTION

In recent decades, there has been a massive evolvement in the nuclear medicine field with the advent of new integrated functional imaging modality, Positron Emission Tomography/Computed Tomography (PET/CT). ^18^F-Fluorodeoxyglucose (FDG) is the most commonly used tracer for PET/CT imaging, especially on cancer diagnosis. Unfortunately, the uptake of FDG has been found to be non-exclusive to malignant tissues alone. Previous report has found that lesions with high concentration of inflammatory cells, such as neutrophils and activated macrophages, will show an incremental uptake of FDG, which potentially can result in false-positive interpretation [1].

Routine diagnostic work-out for spine infection commonly employs a multimodality imaging approach; in particular, magnetic resonance imaging (MRI) and nuclear medicine studies including FDG PET [2, 3].

Current FDG PET/CT protocol for infectious/inflammatory investigation is the same as oncology cases [4]. To the authors’ knowledge, Dual Time Point Imaging (DTPI) using FDG in PET/CT for tuberculous spondylodiscitis with psoas abscess has not been previously described. The aim of this study was therefore to investigate the changes occurring over these two time points.

## CASE REPORT

A 26-year-old woman presented with history of backache for the past several months and increasing in severity. This was associated with low-grade fever and lethargy. Routine physical examination and investigations including total white count, sputum test and urine examination were all negative for tuberculosis (TB) infection, and there was no evidence of lung infections. Other imaging studies including the ultrasonography examination demonstrated that there was an abscess found in her left psoas muscle. Whole body FDG PET/CT examination was done to localise the lesion and identify the extension of the infection. She was confirmed for TB through response to anti-TB treatment where there was a complete metabolic remission upon repeating follow-up PET/CT scanning after six months following completion of the treatment.

## PET/CT STUDY

A DTPI approach was applied during the scanning session. All imaging studies were performed on a dual-modality PET/CT system (Biograph 2, Siemens Medical Solutions^®^). Transmission and emission imaging (Examination 1) started 58 min after intravenous injection of 336.7 MBq (equivalent to 9.1 mCi) of FDG. Total acquisition time for the initial whole body PET/CT was about 30 min. Using the same parameters, a delayed PET/CT imaging (Examination 2) was obtained about 128 min after FDG injection (about 2 hours delay).

## IMAGE ANALYSIS AND SEMI-QUANTITATIVE EVALUATION

PET image datasets were reconstructed iteratively using CT data sets for attenuation correction calculation, creating fusion PET/CT image sets. All co-registered images were reviewed on a workstation in transaxial, coronal and sagittal planes along with Maximum Intensity Projection (MIP) images. The CT images were set in the soft tissue window setting as for general image interpretation. These image analyses were visually and semi-quantitatively interpreted by two experienced nuclear medicine physicians. The evaluating physicians were aware of the patients’ clinical history, including the results of radiological examinations.

The degree of FDG uptake activity in the lesion was visually scored using a 4-point grading system: normal background uptake (Grade 1), mild uptake as in liver or spleen (Grade 2), higher uptake than liver or spleen (Grade 3) and very high uptake with equal or more than brain/bladder uptake (Grade 4). Grade 3 or 4 was considered to represent significantly high FDG uptake. For semi-quantitative analysis, a volume of interest (VOI) was carefully drawn around the site of suspected lesions using the transaxial images. The size of FDG avid lesions was measured on high-resolution CT scan images. For the comparison of initial and delayed images, both images were displayed simultaneously using the same windowing and levelling.

## RESULTS

**Figure 1:**
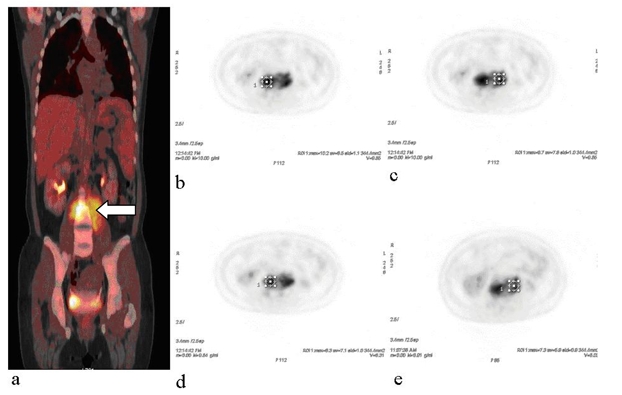
(a) Coronal plane of fusion PET/CT image; showing avid FDG uptake at L3-L4 region affecting the left paravertebral muscle (white arrow); (b) and (c) axial PET images of Examination 1; (d) and (e) axial PET images of Examination 2.

On fusion PET/CT images, there was a huge area detected with high FDG uptake found at lumbar vertebrae region. This finding was highly suspected to be a tuberculous spondylitis with psoas muscle involvement (Figure 1a). The SUVmax values were obtained on axial PET image (Figures 1b to 1e). For third lumbar-fourth lumbar (L3-L4) intervertebral disc and psoas muscle, the SUVmax1 values were 10.2 and 9.7, respectively, whereas, both lesions were declined on delayed imaging i.e. SUVmax2 were 8.3 and 7.3.

## DISCUSSION

FDG PET/CT was performed on a confirmed tuberculous spondylodiscitis patient; at the level of her lumbar region. Based on the authors’ observation, the intensity of FDG uptake of the lesions changed with time following injection. Several studies have reported that tuberculosis can produce high FDG uptake during PET scanning [5, 6], which potentially can cause false positive findings. In overcoming the difficulties in the diagnosis of spine infections, FDG PET offers better imaging features as compared with other imaging modalities. It was possible to clearly differentiate between infections of vertebrae and adjacent soft tissue infections by using FDG PET [2, 3]. Furthermore, this modality is also capable of demonstrating the extent of the infection [2].

DTPI protocol allows comparison between the two sets of images acquired at two different points in time. The characteristic appearance of the lesion and the differences on the intensity of FDG uptake within the studied lesion were recorded. SUVmax values were used to estimate these differences as DTPI protocol has been utilised by others [7, 8]. A recent study by Suga et al. [8] reported that the combination of early SUVmax of more than 3.0 or delayed SUVmax of more than 4.0 was an optimal parameter in differentiating metastatic and benign lymph nodes of the thoracic region. This finding was supported by the fact that FDG uptake in malignant tumours was increased up to several hours after the injection time, and vice versa for benign lesions [9]. Therefore, the utilisation of DTPI protocol may improve the sensitivity and specificity of PET/CT imaging modality [7].

The SUVmax values of the lesions in this case showed a relatively high FDG uptake, up to 10.2 during the first scan. A previous study also found that the SUVmax value of a positive tuberculous spondylodiscitis was as high as 18.1 [3]. In an earlier animal study, Yamada et al. [1] found that inflammatory tissue gradually reached peak FDG uptake for about 60 min after injection, slowly decreasing over time. Therefore, the high FDG uptake value during the first scan with slight reduction on second scan in this case is predicted.

In conclusion, the results of the present study provide further evidence that DTPI protocol of FDG PET/CT appears to be a useful and non-invasive method in the detection of tuberculous spondylodiscitis. Further investigation in a larger group of patients is warranted.
